# Seroprevalence of Sheep and Goat Pox, Peste Des Petits Ruminants and Rift Valley Fever in Saudi Arabia

**DOI:** 10.1371/journal.pone.0140328

**Published:** 2015-10-13

**Authors:** Hani Boshra, Thang Truong, Shawn Babiuk, Maged Gomaa Hemida

**Affiliations:** 1 Canadian Food Inspection Agency, National Centre for Foreign Animal Disease, Winnipeg, Manitoba, Canada; 2 Department of Immunology, University of Manitoba, Winnipeg, Manitoba, Canada; 3 Department of Microbiology and Parasitology, College of Veterinary Medicine and Animal Resources, King Faisal University, Al-Hasa, Saudi Arabia; 4 Department of Virology, Faculty of Veterinary Medicine, Kafrelsheikh University, Kafrelsheikh, Egypt; The University of Texas Medical Branch, UNITED STATES

## Abstract

Sheep and goat pox, peste des petits ruminants and Rift Valley fever are important diseases of small ruminant livestock. Sheep and goat pox, along with peste des petits ruminants, are endemic throughout most of Africa, Asia and the Middle East. Whereas Rift Valley fever is endemic in Africa, outbreaks in the Middle East have been reported over the past decade, including the Arabian Peninsula. Saudi Arabia is a major importer of livestock, and understanding the prevalence of these viral infections would be useful for disease control. In this study, sera from sheep and goats were collected from 3 regions in Saudi Arabia. They were evaluated for antibodies specific to sheep and goat pox, peste des petits ruminants and Rift Valley fever by virus neutralization assays. To the best of our knowledge, this is the first study to evaluate the seroprevalence of these viruses in sheep and goats.

## Introduction

Livestock diseases continue to pose a major threat to the agricultural industry, as well as the world food supply at large [1]. Small ruminant livestock such as sheep and goats are susceptible to sheep pox virus (SPPV), goat pox virus (GTPV), peste des petits ruminants virus (PPRV) and Rift Valley fever virus (RVF). The latter is of particular concern, as it is a vector borne disease that primarily affects ruminants, with past outbreaks resulting in hundreds of human fatalities [2,3,4]. Sheep and goat pox viruses (SPPV and GTPV) are members of family *Poxviridae*, genus Capripoxvirus; and as their name suggests, appear to be specific for sheep and goats, respectively; however, some isolates have been shown to affect both hosts [5]. The disease is characterized by fever and the formation of macules in the skin, which ultimately develop into pox lesions, and can affect over half of the skin surface of affected animals [6]. While the mortality rate is highly variable [ranging from 5–10% in local breeds in endemic regions, to 100% of exotic sheep and goat species in endemic regions [7]], SPPV and GTPV outbreaks have become a major concern in Northern and Central Africa, the Middle East and most of the Asian continent [8,9].

Peste des petits ruminants virus (PPRV) is a *Morbillivirus* [10], which primarily affects small ruminants and is transmitted primarily through direct contact with infected animals through interactions with infected mucosal or fecal secretions [11]. With a mortality rate of 70–80% within 10–12 days of infection, peste des petits ruminants was originally described in the 1940’s in western Africa [12] and currently is endemic throughout Africa except Southern Africa, the Middle East and most of Asia including China [13,14].

Rift Valley fever virus (RVFV) is a member of the *Bunyaviridae* family of RNA viruses [2,3,15]. Rift Valley fever infects a wide range of ruminants including sheep, goats, cattle and camels [16]. In livestock, symptoms range from diarrhea and fever, to widespread abortions in ewes, with widespread death within 24–48 hours [17]. Furthermore, unlike GTPV/SPPV and PPRV, RVFV poses an additional threat to public health, as it is a zoonotic pathogen with a human fatality rate ranging from 1–5% [18]. Originally described by Daubney in the 1930’s in Kenya [19], RVFV outbreaks were limited to sub-Saharan Africa until the 1970’s, when an outbreak in Egypt caused between 20,000–40,000 human cases, and over 600 deaths [20].

These diseases continue to threaten livestock industries in countries where they are endemic, and threaten regions that are free of these diseases with the constant risk of these diseases spreading into new regions. A stark example of this would be the spread of Rift Valley fever in both Yemen and Saudi Arabia from August to October 2000. During that outbreak, hospitals in Saudi Arabia reported 516 cases of the disease with 87 fatalities [21]. Not only did this signal the most severe case of RVF in decades, but more importantly, this marked the first time that this disease was observed outside the African continent.

As previously mentioned, the Arabian Peninsula is currently the only region beyond the African continent to have had all aforementioned livestock diseases (i.e. GTPV/SPPV, PPRV and RVFV). This is of particular concern, since the Middle East serves as a gateway to Europe and Asia. Saudi Arabia serves as a major center for international trade, where hundreds of thousands of small and large ruminants and camels are imported every year to Saudi Arabia for the purpose of slaughtering during the pilgrimage season. This trade in animals provides a significant risk of livestock disease transfer; and in the case of RVFV, the possibility of transferring a zoonotic disease to millions of international travelers [20]. Therefore, while previous risk assessments have been performed for Rift Valley fever in Saudi Arabia (Chevalier et al., 2010), continued surveillance is necessary for monitoring the potential threat of these livestock diseases to the Arabian peninsula, and ultimately, to the international community at large.

Currently, there is a lack of up-to-date information about the prevalence of these viruses in Saudi Arabia. The major goal of the current study was to evaluate the current immune status of several small ruminant flocks against GTPV/SPPV, PPRV and RVFV in Saudi Arabia. Due to the high degree of livestock importation in Saudi Arabia, disease prevalence among these animals can change in a short period of time. To that end, the results obtained in this study will be compared to the limited work previously performed in these areas, in an attempt to determine if any significant change in the presence of these diseases has occurred over the past decade.

## Materials and Methods

### Sera

The sera used in this study were collected according to the Guidelines for Ethical Conduct for Use and Care of Animals in Research of King Faisal University, Saudi Arabia and approved by the King Faisal University ethics care committee. Samples were collected from research projects belonging to the Veterinary hospital of the King Faisal University, Saudi Arabia. Samples from other locations were collected from some private farms in which the owner’s permissions were obtained prior to the collection. Blood collection was done by trained veterinarians and in the presence of the owner of these animals and under his supervision. Blood samples were collected from the jugular vein using the suitable gauge needle as instructed by the above mentioned animal use protocols which as regularly revised and checked before issuing the permission to carry out the designed animal experiments.

Three Saudi Arabian regions representing different parts of the country: Riyadh, Asir and Al-Hasa were selected since they have been shown to have either experienced outbreaks of these diseases, or share borders with other endemic regions. A total of 821 sheep and goat samples were collected from 16 different sites; 3 sites from the eastern province of Al-Hasa, 11 from the central Riyadh region, and 2 sites from the southwestern Asir region. In the case of samples collected from Al-Hasa, sera were collected from 2007, while sera collected from Riyadh and Asir were collected from 2013–2014. Collection was randomized, and included both male and female animals. The 821 samples were analyzed for presence of antibodies against SPPV/GTPV, 780 for PPRV, and 685 for RVFV. It should also be noted that no livestock vaccination campaigns against SPPV/GTPV, PPRV or RVFV were known to have been practiced for the animals under study.

### Virus neutralization assays

Virus neutralizing antibodies were measured for SPPV/GTPV, PPRV and RVFV.

For PPR, serial dilutions (from 1:20 to 1:20,480) of sheep and goat sera, were evaluated by virus neutralization testing (VNT), as previously described [22,23]. Briefly, PPRV (Malig-Yemen strain, 100 TCID_50_, in DMEM) was mixed with sheep and goat sera (in duplicate) to a volume of 200 μl, and incubated for 1 hour at 37°C. Vero cells incubated in 96-well plates were infected with the 200 μl mixture of virus and serum (or DMEM cell media, as a negative control). The cells were then incubated for 15 to 18 days, with daily examination for cytopathic effect (CPE) to evaluate virus neutralization activity. Samples showing inhibition in the CPE at a 1/20 dilution were considered positive.

In the case of sheep and goat pox (SPPV/GTPV), serial dilutions (from 1:10 to 1:10,240) of sheep and goat sera were evaluated. The Kenyan vaccine strain of SPPV (100 TCID_50_, in DMEM) was mixed with sheep and goat sera (in duplicate) to a volume of 200 μl, and incubated for 1 hour at 37°C. OA3.Ts cells were incubated in 96-well plates and were infected with the 200 μl mixture of virus and serum (or DMEM, as a negative control). Cells were incubated for 6–7 days, with daily examination for CPE. A virus neutralizing titre was determined to be the reciprocal of the highest dilution that displayed reduced CPE when compared to a previously characterized negative control. Sample showing inhibition in the CPE at a 1/10 dilution were determined to be positive.

In the case of RVFV, initial screening was performed using sera at a dilution of 1:20 (in duplicate); neutralization assays were performed as previously described using strain ZH501, with minor modifications [24]. Briefly, the diluted sera were incubated with an equal volume of 100 PFU RVFV μl of DMEM at 37°C, and then the sample/virus mixture was added to 100% confluent VeroE6 cells in 48-well plates for 1 hr at 37°C. Following incubation, CMC overlay was added to all cells and incubated for 5–6 days at 37°C. Plaques were visualized by fixation of the plates with 10% formalin, followed by 0.05% crystal violet staining. Samples inducing 70% reduction in plaque formation (relative to their corresponding negative controls) were determined to be positive. VNT assays were then re-performed on the putatively positive samples using serially diluted samples in order to determine endpoint VNT titres.

## Results

**Fig 1:**
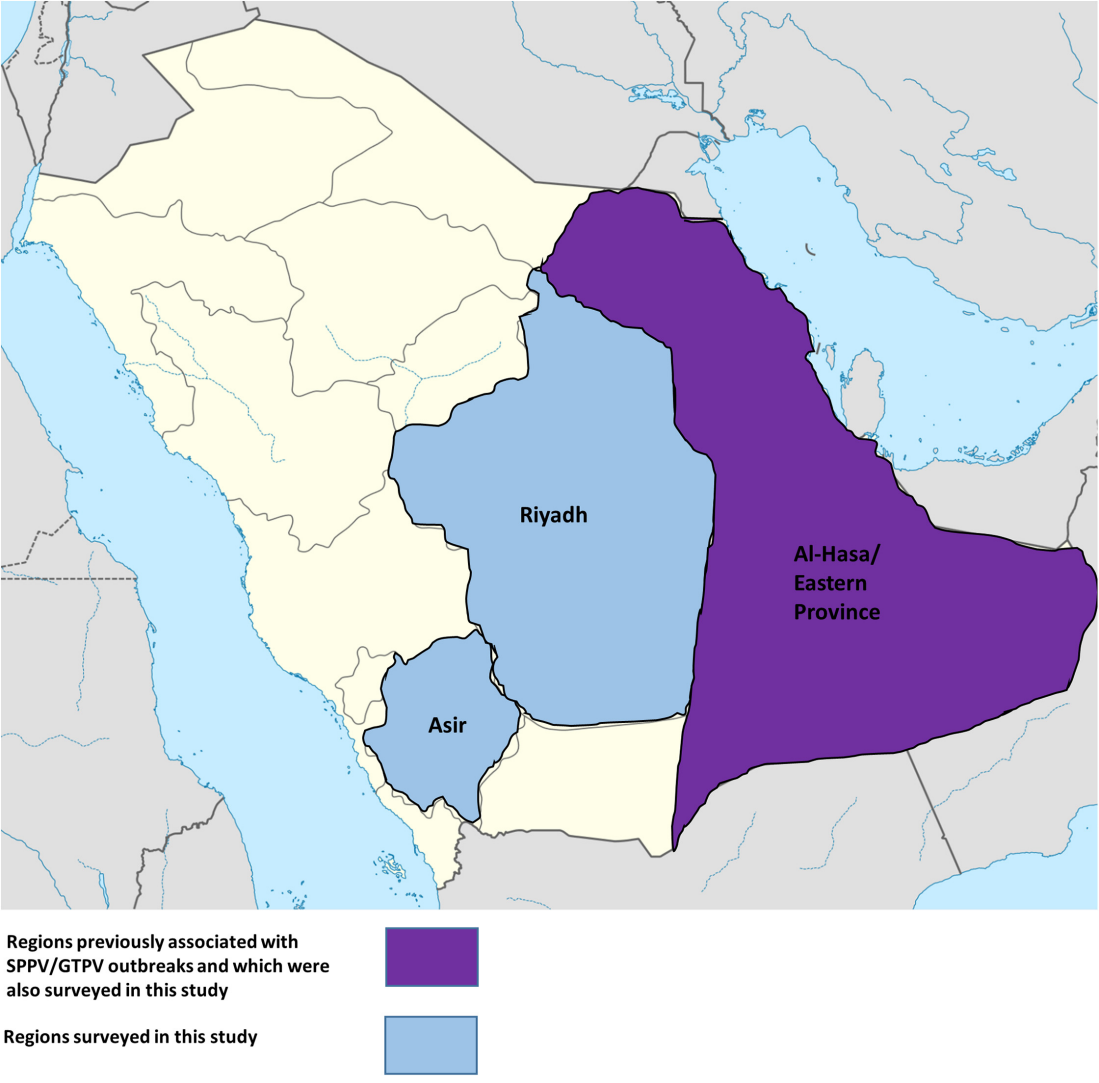
Map of Saudi Arabia, displaying areas to be surveyed for sheep and goat pox (SPPV and GTPV, respectively). Sera were collected from the regions of Asir and Riyadh is indicated in blue. The region to have been previously known to be affected by SPPV and GTPV outbreaks (Al-Hasa/Eastern Province) as well as being one of the regions surveyed in this study is indicated in magenta. Figure has been adapted from original map from NordNordWest.

Serum samples from both sheep and goats in the regions of Asir, Riyadh and the Al-Hasa region of the Eastern Province were evaluated for previous exposure to sheep or goat pox virus (SPPV or GTPV, respectively) (Fig 1). Using VNT as a measure for seroprevalence, it was found that all three regions had animals that were seropositive for SPPV/GTPV (Table 1); however, while both Asir and Al-Hasa had seroprevalence rates of less than 10% (3% and 8%, respectively), the Riyadh region had a rate of more than 42%, indicating a significantly higher presence.

**Table 1:** Seroprevalence of SPPV/GTPV in Saudi Arabian Sheep and Goats.

Region	Year	# of samples	Positive	% positive
Al-Hasa	2007	132	4	3.0
Riyadh	2013–2014	614	259	42.2
Asir	2014	75	6	8.0

**Fig 2:**
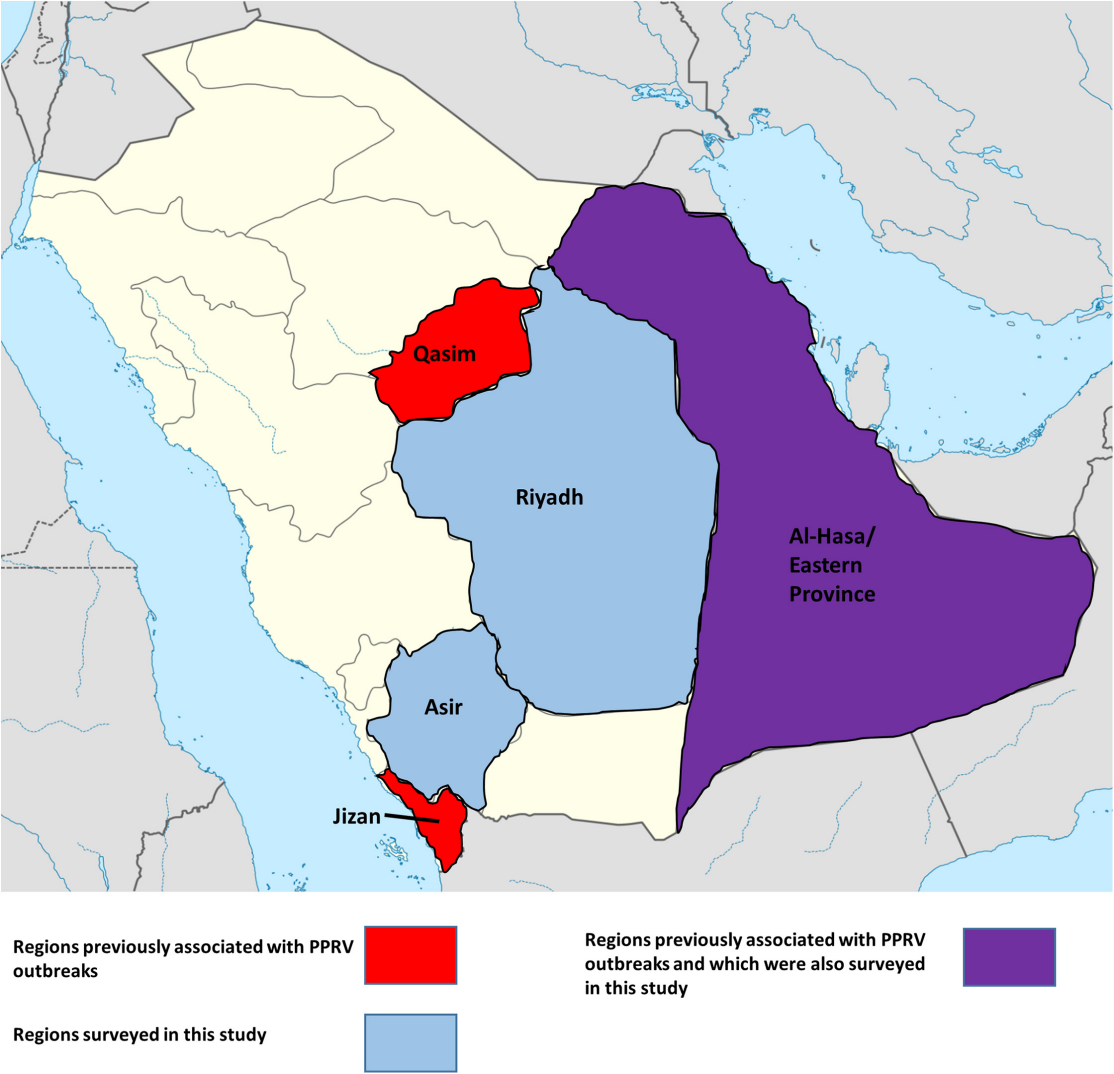
Map of Saudi Arabia, displaying areas to be surveyed for peste des petits ruminants virus (PPRV). Sera were collected from the regions of Asir and Riyadh is indicated in blue. The regions to have been previously known to be affected by PPRV outbreaks (Qasim and Jizan) are indicated in red. In the case of Al-Hasa/Eastern Provence, this region was previously affected by PPRV as well as being one of the regions surveyed in this study is indicated in magenta. Figure has been adapted from original map from NordNordWest.

Interestingly, the high level of seroprevalence in the Riyadh region was not only limited to sheep and goat pox. These same regions were also surveyed for the presence of peste des petits ruminants virus (PPRV) exposure in both sheep and goats. As shown in Fig 2, previous outbreaks of PPR have been documented in the Al-Hasa, as well as Qassim and Jizan regions. As seen in Table 2, while the Riyadh region had the greatest presence of PPRV of all surveyed regions (86%), both the Al-Hasa and Asir regions also had significant levels of seropositive animals (38% and 79%, respectively).

**Table 2:** Seroprevalence of PPRV in Saudi Arabian Sheep and Goats.

Region	Year	# of samples	Positive	% positive
Al-Hasa	2007	133	49	37.7
Riyadh	2013–2014	582	501	86.1
Asir	2014	68	54	79.4

**Fig 3:**
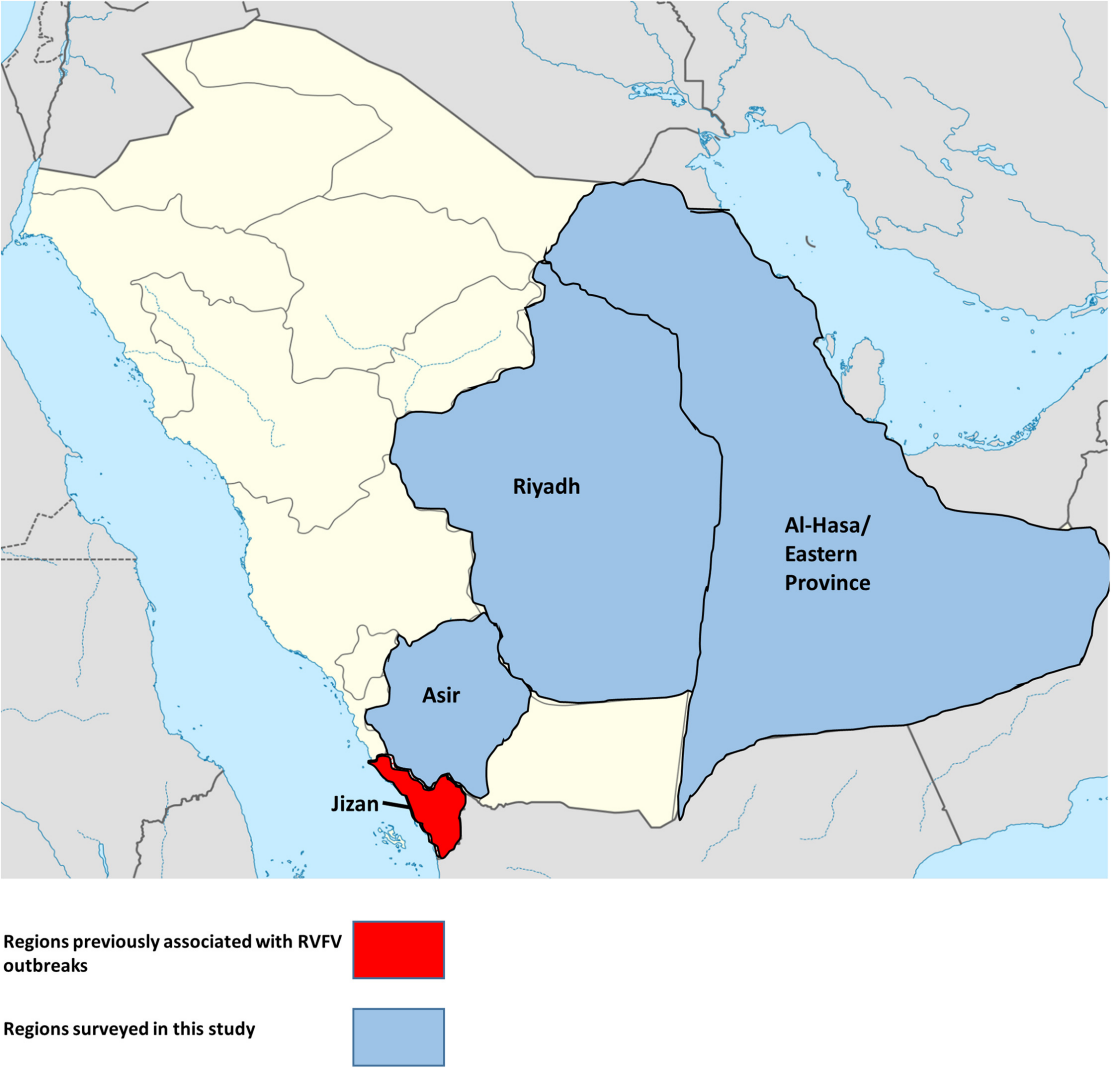
Map of Saudi Arabia, displaying areas to be surveyed for Rift Valley fever virus (RVFV). Sera were collected from the regions of Asir, Riyadh and Al-Hasa (Eastern Province) is indicated in blue. The region to have been previously known to be affected by RVFV outbreaks (Jizan) is indicated in red. Figure has been adapted from original map from NordNordWest.

Finally, serology was also performed from animals in these same regions, using virus neutralization testing (VNT) for the presence of Rift Valley fever virus antibodies (Fig 3). As seen in Table 3, the presence of RVFV antibodies was significantly lower than that for SPPV/GTPV and PPRV. At a 1/20 serum dilution, only a single positive sample was found in both the Riyadh and Asir regions, while the Al-Hasa region had no seropositive samples.

**Table 3:** Seroprevalence of RVFV in Saudi Arabian Sheep and Goats.

Region	Year	# of samples	Positive	% positive
Al-Hasa	2007	118	0	0
Riyadh	2013–2014	509	1	0.2
Asir	2014	58	1	1.7

With the prevalence of RVFV being found to be significantly lower than PPRV and SPPV/GTPV, analysis was then performed to determine if PPRV and SPPV/GTPV displayed any degree of co-localization in all three regions. As seen in Table 4, the proportion of animals that was seropositive for both PPRV and SPPV/GTPV was less than 10% in both Al-Hasa and Asir (1.5% and 7.4%, respectively); while the prevalence of both viruses in Riyadh was more than 39%.

**Table 4:** Seroprevalence of Saudi Arabian Sheep and Goats that are positive for both PPRV and GTPV/SPPV.

Region	Year	# of samples	Positive	% positive
Al-Hasa	2007	130	2	1.5
Riyadh	2013–2014	582	227	39.0
Asir	2014	68	5	7.4

## Discussion

Sheep and goat pox (SPPV and GTPV) outbreaks have been extensively described over the past 50 years, and have been shown to extend from Northern Africa to the Middle East, to as far as India, China and Mongolia [1,8]. While SPPV and GTPV have been suspected of being present in the Arabian Peninsula for at least several decades, it was only during a 1999 outbreak in Saudi Arabia that an isolate of GTPV was isolated and characterized as virulent [25]. Since then, very few studies on the seroprevalence of SPPV and GTPV have been performed in the region. Serosurveillance studies in countries such as Saudi Arabia are particularly problematic, since the traditional and nomadic rearing of sheep and goats makes a detailed inventory of the overall presence of these animals difficult to estimate. Furthermore, as traditional and nomadic rearing of ruminants continues to contribute significantly to the overall production of livestock in the Arabian Peninsula; problems in determining the presence of livestock diseases are even further compounded. A study was conducted on sheep populations in the Eastern Province in 2013, following the appearance of pox-like symptoms in multiple flocks of sheep in the region, with morbidity and mortality rates of affected animals approaching 80% and 15%, respectively [25]. Using molecular diagnostic techniques, the causative agent was found to be a strain of SPPV that shared phylogenetic homology to SPPV isolates from China and India, thereby confirming the presence of both in SPPV and GTPV in the region.

In the present study, we used VNTs to further monitor the seroprevalence of these capripoxviruses in the Eastern Province (Al-Hasa), as well as in the central Riydah and southwestern Asir regions, in order to evaluate the extent to which these capripoxviruses are present (Fig 1). As shown in Table 1, some sheep and goats in the Al-Hasa region did have neutralizing antibodies against capripox, but ultimately had a seroprevalence rate of less than 4%. While the outbreak of 2013 in Al-Hasa led us to expect that some animals would show evidence of capripox exposure, the significantly high levels of positive samples from the Riyadh region was unexpected, as no official outbreaks have been reported, nor have any vaccine campaigns been known to have been carried out in the flocks under study. The lack of any previous surveys in the area makes it difficult to conclude whether the presence of capripox in these areas is a result of a longtime presence of the virus in the region, or is the result of a recent outbreak caused by an introduction through the migration or trade of livestock. Therefore, regular serosurveillance will be necessary to determine the dynamics of capripox infection in the Arabian Peninsula.

In the past 30 years, outbreaks of peste des petits ruminants have been described outside of Western Africa. In the 1980’s PPR was reported in sheep and goats in Sudan. Since then, PPRV outbreaks have continued eastward, and have been reported to affect livestock as far as India, as well as the Tibetan region of China. During the course of its spread, multiple outbreaks of the disease have been reported in the Arabian Peninsula. During the late 1980’s suspected cases of PPR were being observed in sheep, gazelles and deer [26]; this included a severe outbreak of the disease in the Al-Hasa region in 1988 [27] with mortality rates approaching 70%. Since then, another outbreak that affected two flocks in the Jizan region of Saudi Arabia was reported in 2002 [28], with herd mortality rates approaching 100% [29]. Another outbreak was reported in Al-Qassim region in central Saudi Arabia in 2005, with morbidity and mortality rates being approximated at 20% and 3%, respectively [30]. In this study, the tested sera from flocks from the Asir region were collected nearly 5 years after the nearby Jizan outbreak; while samples from the Al-Hasa region were collected nearly 20 years after the first reported outbreak in that area. Samples were also collected from the Riyadh region, south of the Qassim region (Fig 2).

The presence of PPRV specific antibodies from animals in all three regions was significant, ranging from nearly 40% in the Al-Hasa region (from 2007), to nearly 80% in Asir and 85% in Riyadh (from 2013–3014). The latter of the three is of particular interest, since a previous survey of the nearby Al-Qassim region following the 2005 PPRV outbreak found sheep and goat seroprevalence to be 37% and 55%, respectively [30]. Considering animals in the region have not been vaccinated, it can be concluded that widespread seroprevalence is likely due to: 1) continued transmission within local sheep and goat flocks; 2) the introduction of PPRV to livestock through interaction with wild animal reservoirs, or 3) continuous introduction of these viruses from imported animals from Africa. In the first scenario, continued transmission of PPRV within livestock populations may be possible due to the low incidence of mortality related to a particular strain (as evidenced by the low mortality rate of the previous 2005 outbreak). The latter possibility of external transmission may also be of particular significance, since it has been suggested that transmission of PPRV from camels to goats may be possible [31]. It should also be noted that gazelles have also been found to carry PPRV [32], and may also be a factor in the transmission of the disease among livestock.

As both SPPV/GTPV and PPRV were significantly higher than RVFV, the degree of co-localization of carpripox and PPRV in the sheep and goats of these regions was then analyzed. As seen in Tables 1 and 4, the rate of SPPV/GTPV infection alone appears to be similar to the prevalence of both SPPV/GTPV and PPRV. This may suggest that livestock with previous exposure to capripox may have an increased susceptibility to PPRV infection, as suggested in previous studies [33]; however, due to the high overall prevalence of PPRV, as well as the limited information on the outbreaks in these regions, such a hypothesis cannot be confirmed to any statistical significance.

The outbreak of RVFV in Saudi Arabia and Yemen in 2000 marked a significant milestone in the transmission of the disease, as it marked the first time that that such an outbreak was observed outside of Africa. Although zoonotic, initial responses to the 2000 outbreak placed particular emphasis on human transmission, as the severity of the disease led to nearly 900 cases from November 2000 to September 2001 [34]. One of the earliest surveys in livestock was described in 2006 [35]. In that study, ELISA assays was used to detect RVFV IgG on more than 3000 sheep and goats samples in the Jizan region (which is of particular interest, since this region was also the site of the original 2000 outbreak). In that study, nearly 30% of the tested animals were positive for RVFV IgG. In 2012, serosurveillance of RVFV [36], revealed that only 4 out of more than 2400 animals (spanning all regions of Saudi Arabia, including Jizan) had virus-specific IgG. This represented a significant decline in small ruminants that were exposed to the virus. However, this decline may also be due to methodology, as a 2011 RVFV survey of 500 ruminants in a Mecca slaughterhouse showed that over 16% of sacrificial sheep and goats were seropositive using competitive ELISA [37].

In this study, we tested almost 700 sheep and goats samples from three areas spanning Saudi Arabia; Riyadh, Asir and Al-Hasa. Samples from Al-Hasa were collected from 2007, while samples from Riyadh and Asir were collected from 2013–2014. As seen in Fig 3, of the three regions surveyed, the Asir region was of particular interest, since outbreaks of Rift Valley fever have previously been reported in the adjacent Jizan region over a decade ago. Despite the fact that Asir was also associated with the initial outbreak in 2000, the lack of any significant positive samples from that region, as well as the other three regions, were consistent with the results from Al-Afaleq *et al* (2012). In our study, only 2 samples were found to be positive; furthermore, it should be noted that the titers for both positive samples did not exceed 1/20. Therefore, it is possible that the VNT observed for both samples may be non-specific.

All three studies suggest that, while prevalent in the years immediately following the 2000 outbreak, the lack of any significant presence of RVFV in sheep and goat over the following decade is consistent with the lack of outbreaks during the same period. However, it should be noted that in 2010, a small outbreak of RVFV occurred in the Jizan region, affecting cattle, sheep and goats [38]. The limited scope of this outbreak could be due to either latent infected mosquito eggs in the region, or the limited introduction of RVFV-infected mosquitos from an outbreak in Kenya that occurred several months prior.

While this study did not have any samples from that individual outbreak, the lack of a large number of seropositive samples from the nearby Asir region suggests that the outbreak was either limited in scope, or effectively contained immediately after detection.

Finally, the sporadic detection of significant seropositive samples from sheep and goat are consistent with the nature of RVFV epidemiology. As previously mentioned, RVFV outbreaks rely on several factors, including excessive rainfall, the creation of novel irrigation systems, as well as the natural migration of mosquito vectors. Previous studies have already implicated mosquitos that are already indigenous in Saudi Arabia (i.e. *Culex tritaeniorhynchus* Giles) in the previous Jizan outbreak [39,40]. All these factors, in turn, make the prediction of new outbreaks difficult. Therefore, continued screening and surveillance for RVFV will be necessary in order to limit future spread of this pathogen. Furthermore, more extensive studies on local arthopod vectors, as well as the means of RVFV transmission will be necessary in establishing a basal level of RVFV epidemiology and immunosurveillance.
